# An Optimal Mean Based Block Robust Feature Extraction Method to Identify Colorectal Cancer Genes with Integrated Data

**DOI:** 10.1038/s41598-017-08881-3

**Published:** 2017-08-17

**Authors:** Jian Liu, Yuhu Cheng, Xuesong Wang, Lin Zhang, Hui Liu

**Affiliations:** School of Information and Control Engineering, China University of Mining and Technology, Xuzhou, 221116 China

## Abstract

It is urgent to diagnose colorectal cancer in the early stage. Some feature genes which are important to colorectal cancer development have been identified. However, for the early stage of colorectal cancer, less is known about the identity of specific cancer genes that are associated with advanced clinical stage. In this paper, we conducted a feature extraction method named Optimal Mean based Block Robust Feature Extraction method (OMBRFE) to identify feature genes associated with advanced colorectal cancer in clinical stage by using the integrated colorectal cancer data. Firstly, based on the optimal mean and *L*_2,1_-norm, a novel feature extraction method called Optimal Mean based Robust Feature Extraction method (OMRFE) is proposed to identify feature genes. Then the OMBRFE method which introduces the block ideology into OMRFE method is put forward to process the colorectal cancer integrated data which includes multiple genomic data: copy number alterations, somatic mutations, methylation expression alteration, as well as gene expression changes. Experimental results demonstrate that the OMBRFE is more effective than previous methods in identifying the feature genes. Moreover, genes identified by OMBRFE are verified to be closely associated with advanced colorectal cancer in clinical stage.

## Introduction

Colorectal cancer which is also known as bowel cancer, colon cancer, or rectal cancer is the development of cancer in the colon, rectumor parts of the large intestine. Globally, colorectal cancer is the 3rd most common cancer, which account for about 10%. There were about 1.4 million new occurrences and 694,000 deaths from colorectal cancer each year^1^. It is more common in developed countries, e.g., the five year survival ratesof the disease are around 65% in the United States. It, however, depends on how early the colorectal cancer is diagnosed^2^.

Recently, some feature genes that are important to colorectal cancer progression have been identified based on the development in genetics and genomics research^3–7^. For example, the cancer genes APC and KRAS are known to play important roles in colorectal cancer due to the high frequency of genetic aberrations in colorectal cancer^7^. Though these cancer genes have been characterized to be related to colorectal cancer development directly, for the early stage of colorectal cancer, less is known about which genes are closely associated with the progressive stage.

Clinically, colorectal cancer can be treated by surgical resection. Nevertheless, the recurrence and metastasis of colorectal cancer still occur frequently even if the tumor has been curatively resection successfully since the cancer is a metastatic disease^8^. The metastasis status of colorectal cancer is a main factor leading to the increased mortality of patients and is assessed to depend on the clinical stage. Advanced clinical stage of colorectal cancer can either reflect metastatic cancer spread to the regional lymph nodes around the colon or spread to organs outside the colon or rectum. Compared to the early stage of colorectal cancer which is generally considered to be cured, the advanced clinical stage has a significantly worse prognosis. Hence, identification of the feature genes associated with advanced clinical stage of colorectal cancer may illuminate the underlying genetics and contribute to the prognostic assessment^9^.

Recently, many feature extraction algorithms have been put forward in the field of biological information processing to identify differentially expressed genes. Among these methods, singular value decomposition (SVD) and principal component analysis (PCA)^10^ are most commonly used for dimensionality reduction and feature extraction. However, the *L*_2_-norm based objective function makes the method sensitive to data outliers. The data outliers always prevalently exist in datasets and thus affect the performance of algorithms. Hence, SVD and PCA cannot obtain the optimal performance due to their *L*_2_-norm based objective function. To address this issue, multiple methods have been proposed, wherein *L*_1_-norm and *L*_2,1_-norm are the most widely used solution. *L*_1_-norm minimization is a convex optimization problem which can reduce the effect of data outlier. Up to now, *L*_1_-norm is applied to many feature extraction algorithms. For instance, in penalized matrix decomposition (PMD) method which is implemented by using SVD, *L*_1_-norm was considered as the penalty function to obtain the optimal solution^11^; in robust principal component analysis (RPCA) method, *L*_1_-norm was taken to improve the robustness of the algorithm^12^. Moreover, both PMD and RPCA methods are applied to extract feature genes successfully^13, 14^. Ding *et al*. proposed the rotational invariant L1PCA by imposing *L*_2_-norm on the feature and *L*_1_-norm on the data points in order to minimize the *L*_2,1_-norm reconstruction error^15^.

Though these methods can achieve relatively better performances, they still have some shortcomings. One disadvantage is that all these methods neglect the mean calculation problem. Because in different robust methods, the Euclidean distance based mean is not the correct one while the *L*_1_-norm or the *L*_2,1_-norm is utilized as the loss function. Nie *et al*. put forward the optimal mean RPCA method by removing the optimal mean automatically^16^.

In this paper, in view of the optimal mean in [16], we propose a novel feature extraction method called Optimal Mean based Robust Feature Extraction (OMRFE) method by using SVD to identify feature genes. In our method, the data matrix ***X*** is decomposed into two full rank matrices ***W*** and ***V*** ^*T*^ by SVD. The critical information of the data matrix ***X*** can be captured by ***W*** ^14^. Therefore, the feature genes can be identified by optimizing ***W***.

Conventional feature extraction methods, such as PMD, RPCA, even OMRFE, are quite effective in processing gene expression data. However, in some cases these methods are not applicable, for instance, for the datasets provided by TCGA, multiple genomic features are usually integrated into one dataset for some purposes, which may make the conventional feature extraction methods unreasonable since conventional feature extraction methods can only process single type of genomic feature. Thus, a novel method to handle the integrated TCGA datasets should be studied.

The Cancer Genome Atlas (TCGA) genomic dataset provides an opportunity to consider different categories of genetic aberrations in gene resolution^17–19^. The combination of multiple genomic features can improve the prediction accuracy comparing to an individual genomic feature^20, 21^. Based on the TCGA colorectal cancer data, Lee *et al*. integrated multiple classes of available genomic data, which integrated copy number alterations, somatic mutations, methylation and gene expression changes together^9^. We can identify the feature genes associated with advanced colorectal cancer in clinical stage via the integrated data. Since it comprises four different genomic datasets and the distribution of each dataset is different, it is inappropriate to process the integrated data as a single data for conventional methods. Different genomic data should have different constraint parameters, so the block ideology is suitable to deal with the integrated data^22^. Therefore, relying on OMRFE method, we propose another feature extraction method for the integrated colorectal cancer data named the Optimal Mean based Block Robust Feature Extraction (OMBRFE) method. In OMBRFE, multiple regularization parameters are adopted to process the integrated colorectal cancer data.

The main contributions of this paper are described as follows: Firstly, relying on the optimal mean, we proposed a novel feature extraction method OMRFE to identify the feature genes. Secondly, in order to integrate multiple colorectal cancer data, we applied the block ideology to the OMRFE and put forward a new method OMBRFE to identify specific cancer genes associated with advanced colorectal cancer in clinical stage.

The remainder of this study is structured as follows. In Section 2, the methodology of OMRFE and OMBRFE is shown. Then how to identify the feature genes using OMRFE and OMBRFE is introduced. The experimental results and discussion are presented in Section 3. In Section 4, the conclusion is shown.

## Methods

### Optimal mean

Traditionally, many robust PCA methods ignore the mean calculation problem. The *L*_2_-norm distance based mean is not the correct mean when these PCA methods are implemented by *L*_1_-norm or *L*_2,1_-norm. In literature [16], a novel robust PCA is proposed by removing the optimal mean automatically. The optimal mean calculation is integrated into the dimensionality reduction optimization objection for enhancement. Both theoretical analysis and experimental results prove that the optimal mean based robust PCA can more effectively reduce data dimensionality than previous methods^16^. In this paper, optimal mean theory is utilized to identify cancer genes.

Given a data matrix ${\boldsymbol{X}}\in {{\mathbb{R}}}^{m\times n}$, where *m* is the dimensionality and *n* is the number of samples. Generally, SVD is used to find a low-rank matrix which can best approximate the data matrix based on Euclidean distance. SVD is used to solve the following problem:

1 $$\mathop{{\rm{\min }}}\limits_{{\boldsymbol{W}},{\boldsymbol{V}},{{\boldsymbol{W}}}^{T}{\boldsymbol{W}}={\boldsymbol{I}}}{\Vert {\boldsymbol{X}}-{\boldsymbol{W}}{{\boldsymbol{V}}}^{T}\Vert }_{F}^{2}.$$

where ***W*** and ***V*** ^*T*^ are full rank matrices, ${\boldsymbol{W}}\in {{\mathbb{R}}}^{m\times k}$, ${\boldsymbol{V}}\in {{\mathbb{R}}}^{n\times k}$ and ***W*** ^*T*^ ***W*** = ***I***. By setting the derivative w.r.t ***V*** in Eq. (1) to zero, we can obtain ***V*** = ***X*** ^*T*^ ***W***. Thus, Eq. (1) can be solved by:

2 $$\mathop{\max }\limits_{{\boldsymbol{W}},{{\boldsymbol{W}}}^{T}{\boldsymbol{W}}={\boldsymbol{I}}}Tr({{\boldsymbol{W}}}^{T}{\boldsymbol{X}}{{\boldsymbol{X}}}^{T}{\boldsymbol{W}}).$$

Therefore, the optimal solution ***W*** to Eq. (2) can be described as the *k* eigenvectors of ***XX*** ^*T*^ corresponding to *k* largest eigenvalues.

In the above derivation process, the mean of the data matrix is usually supposed to be zero. But in general cases, the mean of the data matrix always does not equal to zero. So we should attempt to best approximate the given data matrix with an optimal mean removed. Denote ${\boldsymbol{a}}\in {{\mathbb{R}}}^{n\times 1}$ as a column vector with all the elements being one and ${\boldsymbol{b}}\in {{\mathbb{R}}}^{m\times 1}$ as a variable to be optimized, then ${\boldsymbol{b}}{{\boldsymbol{a}}}^{T}\in {{\mathbb{R}}}^{m\times n}$ and ${\boldsymbol{X}}\in {{\mathbb{R}}}^{m\times n}$ has the same size. Here, ***ba*** ^*T*^ can be denoted as the mean of the data matrix needing optimization. After removing an optimal mean, Eq. (1) becomes:

3 $$\mathop{\min }\limits_{{\boldsymbol{W}},{\boldsymbol{V}},{\boldsymbol{b}},{{\boldsymbol{W}}}^{T}{\boldsymbol{W}}={\boldsymbol{I}}}{\Vert {\boldsymbol{X}}-{\boldsymbol{b}}{{\boldsymbol{a}}}^{T}-{\boldsymbol{W}}{{\boldsymbol{V}}}^{T}\Vert }_{F}^{2}.$$

Taking the derivative w.r.t ***V*** in Eq. (3) and setting it to zero, we can obtain ***V*** = (***X*** − ***ba*** ^*T*^)^*T*^ ***W***. Then, Eq. (3) can be written as

4 $$\mathop{\min }\limits_{{\boldsymbol{W}},{\boldsymbol{b}},{{\boldsymbol{W}}}^{T}{\boldsymbol{W}}={\boldsymbol{I}}}{\Vert {\boldsymbol{X}}-{\boldsymbol{b}}{{\boldsymbol{a}}}^{T}-{\boldsymbol{W}}{{\boldsymbol{W}}}^{T}({\boldsymbol{X}}-{\boldsymbol{b}}{{\boldsymbol{a}}}^{T})\Vert }_{F}^{2}.$$

Taking the derivative w.r.t ***b*** in Eq. (4) and setting it to zero, we can obtain (***I*** − ***WW*** ^*T*^)(***ba*** ^*T*^ − ***X***)***a*** = 0. Denote the orthogonal complement of ***W*** as ***W*** ^⊥^, the (***ba*** ^*T*^ − ***X***)***a*** can be represented as follows

5 $$({\boldsymbol{b}}{{\boldsymbol{a}}}^{T}-{\boldsymbol{X}}){\boldsymbol{a}}={\boldsymbol{W}}\alpha +{{\boldsymbol{W}}}^{\perp }\beta ,$$

where *α* could be any *k*-dimensional column vector. Thus, we obtain (***I*** − ***WW*** ^*T*^)(***W*** *α* + ***W*** ^⊥^ *β*) = 0. Since (***I*** − ***WW*** ^*T*^)***W*** *α* = ***W*** *α* − ***WW*** ^*T*^ ***W*** *α* = 0, (***I*** − ***WW*** ^*T*^)***W*** ^⊥^ *β* = 0 ⇔ ***W*** ^⊥^ *β* = 0 ⇔ *β* = 0. Then Eq. (5) can be written as

6 $${\boldsymbol{b}}=\frac{1}{n}({\boldsymbol{Xa}}+{\boldsymbol{W}}\alpha ).$$

Suppose ${\boldsymbol{C}}={\boldsymbol{I}}-\tfrac{1}{n}{\boldsymbol{a}}{{\boldsymbol{a}}}^{T}$ is a centering matrix, we substitute Eq. (6) into Eq. (4) and obtain the following form

7 $$\mathop{\max }\limits_{{\boldsymbol{W}},{{\boldsymbol{W}}}^{T}{\boldsymbol{W}}={\boldsymbol{I}}}Tr({{\boldsymbol{W}}}^{T}{\boldsymbol{XC}}{{\boldsymbol{X}}}^{T}{\boldsymbol{W}}).$$

It can be seen that Eq. (7) is changeless whether ***X*** is centered or not. The optimal mean in Eq. (3) is ${\boldsymbol{b}}=\tfrac{1}{n}{\boldsymbol{Xa}}$ with *α* = 0 in Eq. (6). Therefore, the data matrix can be simply centered as ***Xa*** = 0, then the solution of Eq. (7) can be replaced by the solution of Eq. (2).

In many robust algorithms, *L*_2,1_-norm is widely used to improve the robustness. However, the data matrix is still centered by using *L*_2_-norm distance based mean. In [16], Nie *et al*. demonstrated that the Euclidean distance based mean is not the correct one with the *L*_2,1_-norm being the loss function. Then the following problem should be solved

8 $$\mathop{\min }\limits_{{\boldsymbol{W}},{\boldsymbol{V}},{\boldsymbol{b}},{{\boldsymbol{W}}}^{T}{\boldsymbol{W}}={\boldsymbol{I}}}{\Vert {\boldsymbol{X}}-{\boldsymbol{b}}{{\boldsymbol{a}}}^{T}-{\boldsymbol{W}}{{\boldsymbol{V}}}^{T}\Vert }_{2,1}.$$

Eq. (8) can be rewritten as follows:

9 $$\mathop{\min }\limits_{{\boldsymbol{W}},{\boldsymbol{V}},{\boldsymbol{b}},{{\boldsymbol{W}}}^{T}{\boldsymbol{W}}={\boldsymbol{I}}}\sum _{i}^{n}{\Vert {{\boldsymbol{x}}}_{i}-{\boldsymbol{b}}-{\boldsymbol{W}}{({{\boldsymbol{v}}}^{i})}^{T}\Vert }_{2}.$$

Similar to conventional SVD, we can obtain the following formula

10 $$\mathop{\min }\limits_{{\boldsymbol{W}},{\boldsymbol{b}},{{\boldsymbol{W}}}^{T}{\boldsymbol{W}}={\boldsymbol{I}}}\sum _{i}^{n}{\Vert ({\boldsymbol{I}}-{\boldsymbol{W}}{{\boldsymbol{W}}}^{T})({{\boldsymbol{x}}}_{i}-{\boldsymbol{b}})\Vert }_{2}.$$

Eq. (10) can be solved by using an iterative re-weighted method, and the detailed algorithm can be found in [16]. In each iteration, the following problem is solved

11 $$\mathop{\min }\limits_{{\boldsymbol{W}},{\boldsymbol{b}},{{\boldsymbol{W}}}^{T}{\boldsymbol{W}}={\boldsymbol{I}}}\sum _{i}^{n}{d}_{ii}{\Vert ({\boldsymbol{I}}-{\boldsymbol{W}}{{\boldsymbol{W}}}^{T})({{\boldsymbol{x}}}_{i}-{\boldsymbol{b}})\Vert }_{2}^{2},$$

where *d*_*ii*_ is the weight. Taking the derivation w.r.t ***b*** and setting it to zero, then (***I*** − ***WW*** ^*T*^)(***ba*** ^*T*^ − ***X***)***Da*** = 0. Following the traditional SVD, we get (***ba*** ^*T*^ − ***X***)***Da*** = ***W*** *α*, then the optimal mean becomes

12 $${\boldsymbol{b}}=\frac{{\boldsymbol{XDa}}}{{{\boldsymbol{a}}}^{T}{\boldsymbol{Da}}}+\frac{{\boldsymbol{W}}\alpha }{{{\boldsymbol{a}}}^{T}{\boldsymbol{Da}}}.$$

We can substitute Eq. (12) into Eq. (11) and obtain the following form

13 $$\mathop{\max }\limits_{{\boldsymbol{W}},{{\boldsymbol{W}}}^{T}{\boldsymbol{W}}={\boldsymbol{I}}}Tr({{\boldsymbol{W}}}^{T}{\boldsymbol{X}}{{\boldsymbol{C}}}_{d}{{\boldsymbol{X}}}^{T}{\boldsymbol{W}}),$$

where ${{\boldsymbol{C}}}_{d}={\boldsymbol{D}}-\tfrac{{\boldsymbol{Da}}{{\boldsymbol{a}}}^{T}{\boldsymbol{D}}}{{{\boldsymbol{a}}}^{T}{\boldsymbol{Da}}}$ is the weighted centering matrix. Therefore, the optimal solution ***W*** to Eq. (13) can be described as *k* eigenvectors of ***XC***_*d*_ ***X*** ^*T*^ corresponding to *k* largest eigenvalues.

### Description of OMRFE

At first, we decompose the matrix ***X*** into two full rank matrices ***W*** and ***V*** ^*T*^ via SVD, ***X*** = ***WV*** ^*T*^.

The general feature extraction problem is always defined as

14 $$\mathop{\min }\limits_{{\boldsymbol{W}},{\boldsymbol{V}},{{\boldsymbol{W}}}^{T}{\boldsymbol{W}}={\boldsymbol{I}}}{\Vert {\boldsymbol{X}}-{\boldsymbol{W}}{{\boldsymbol{V}}}^{T}\Vert }_{F}^{2}.$$

Following [14], the feature genes can be extracted according to ***W***. In order to improve the robustness to outliers, *L*_2,1_-norm is adopted as the loss function

15 $$\mathop{\min }\limits_{{\boldsymbol{W}},{\boldsymbol{V}},{{\boldsymbol{W}}}^{T}{\boldsymbol{W}}={\boldsymbol{I}}}{\Vert {\boldsymbol{X}}-{\boldsymbol{W}}{{\boldsymbol{V}}}^{T}\Vert }_{2,1}.$$

Then we use the nuclear norm to obtain the low rank of ***W***: ${\Vert {\boldsymbol{W}}\Vert }_{\ast }$. And the preliminary feature extraction problem is given as follows:

16 $$\mathop{\min }\limits_{{\boldsymbol{W}},{{\boldsymbol{W}}}^{T}{\boldsymbol{W}}={\boldsymbol{I}}}{\Vert {\boldsymbol{X}}-{\boldsymbol{W}}{{\boldsymbol{V}}}^{T}\Vert }_{2,1}+\lambda {\Vert {\boldsymbol{W}}\Vert }_{\ast },$$

where *λ* is the regularization parameter.

According to the optimal mean ideology in [16], the optimal mean of data matrix ***X*** should be removed, that is ***X*** − ***ba*** ^*T*^. Then the decomposition of ***X*** − ***ba*** ^*T*^ becomes ***X*** − ***ba*** ^*T*^ = ***WV*** ^*T*^. So Eq. (16) should be corrected as

17 $$\mathop{\min }\limits_{{\boldsymbol{W}},{\boldsymbol{b}},{{\boldsymbol{W}}}^{T}{\boldsymbol{W}}={\boldsymbol{I}}}{\Vert {\boldsymbol{X}}-{\boldsymbol{b}}{{\boldsymbol{a}}}^{T}-{\boldsymbol{W}}{{\boldsymbol{V}}}^{T}\Vert }_{2,1}+\lambda {\Vert {\boldsymbol{W}}\Vert }_{\ast }.$$

Since ***X*** − ***ba*** ^*T*^ = ***WV*** ^*T*^, where ***V*** ^*T*^ ***V*** = ***I***, we multiply both sides of the formula by ***V***, then the formula becomes (***X*** − ***ba*** ^*T*^)***V*** = ***W***. For more convenience, Eq. (17) can be easily converted as follows:

18 $$\mathop{\min }\limits_{{\boldsymbol{W}},{\boldsymbol{b}},{{\boldsymbol{W}}}^{T}{\boldsymbol{W}}={\boldsymbol{I}}}{\Vert ({\boldsymbol{X}}-{\boldsymbol{b}}{{\boldsymbol{a}}}^{T}){\boldsymbol{V}}-{\boldsymbol{W}}\Vert }_{2,1}+\lambda {\Vert {\boldsymbol{W}}\Vert }_{\ast }.$$

The optimal result of Eq. (18) can be obtained by using the Augmented Lagrangian Multiplier (ALM) method.

Following the ALM method, we rewrite Eq. (18) as

19 $$\mathop{\min }\limits_{{\boldsymbol{W}},{\boldsymbol{b}},{\boldsymbol{E}},{{\boldsymbol{W}}}^{T}{\boldsymbol{W}}={\boldsymbol{I}}}{\Vert {\boldsymbol{E}}\Vert }_{2,1}+\lambda {\Vert {\boldsymbol{W}}\Vert }_{\ast }+\frac{\mu }{2}{\Vert ({\boldsymbol{X}}-{\boldsymbol{b}}{{\boldsymbol{a}}}^{T}){\boldsymbol{V}}-{\boldsymbol{W}}-{\boldsymbol{E}}+\frac{1}{\mu }{\rm{\Lambda }}\Vert }_{F}^{2},$$

where ***E*** = (***X*** − ***ba*** ^*T*^)***V*** − ***W***, Λ is the Lagrange multiplier, *μ* is a positive scalar. In Eq. (19), there exist three variables ***W***, ***b***, and ***E*** which make the solution very difficult.

Following the alternating method^23^, we fix ***E*** in Eq. (19) and rewrite it as

20 $$\mathop{\min }\limits_{{\boldsymbol{W}},{\boldsymbol{b}},{{\boldsymbol{W}}}^{T}{\boldsymbol{W}}={\boldsymbol{I}}}\frac{\mu }{2}{\Vert ({\boldsymbol{X}}-{\boldsymbol{b}}{{\boldsymbol{a}}}^{T}){\boldsymbol{V}}-{\boldsymbol{E}}+\frac{1}{\mu }{\rm{\Lambda }}-{\boldsymbol{W}}\Vert }_{F}^{2}+\lambda {\Vert {\boldsymbol{W}}\Vert }_{\ast }.$$

Eq. (20) can be solved with the lemmas in [16] to update ***W*** and ***b***. When fixing ***W*** and ***b***, Eq. (19) becomes

21 $$\mathop{\min }\limits_{{\boldsymbol{E}}}\frac{\mu }{2}{\Vert {\boldsymbol{E}}-({\boldsymbol{X}}-{\boldsymbol{b}}{{\boldsymbol{a}}}^{T}){\boldsymbol{V}}+{\boldsymbol{W}}-\frac{1}{\mu }{\rm{\Lambda }}\Vert }_{F}^{2}+{\Vert {\boldsymbol{E}}\Vert }_{2,1}.$$

Eq. (20) can be solved to update ***E*** ^16^.

In summary, the brief algorithm of OMRFE is shown as follows
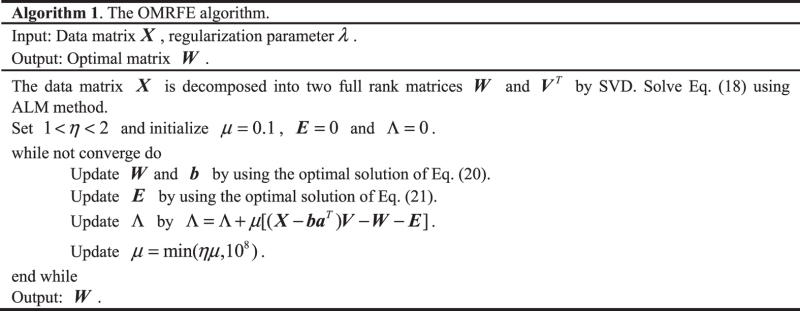


### Identify feature genes using OMRFE

**Figure 1:**
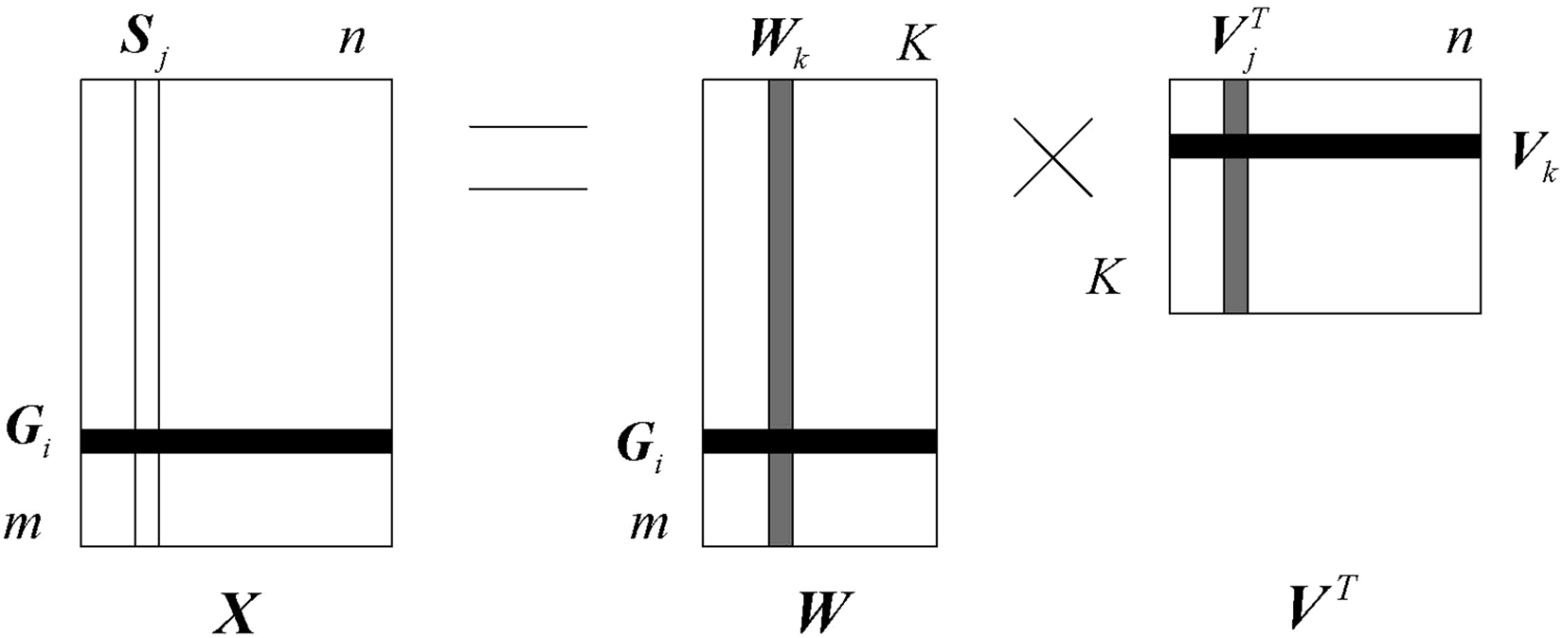
The graphical depiction of gene identification using OMRFE.

We can denote the gene expression data as matrix ${\boldsymbol{X}}\in {{\mathbb{R}}}^{m\times n}$. In ***X***, each row is the expression level of a gene in all *n* samples; each column is the expression level of *m* genes in a single sample. According to the convention in ref. 24, ***X*** can be decomposed into ***W*** and ***V*** ^*T*^ using OMRFE. Fig. 1 shows the graphical depiction of gene identification using OMRFE, where ***G***_*i*_ ($i=1,2,\cdots ,m$) is the gene transcriptional responses, ***S***_*j*_ ($j=1,2,\cdots ,n$) is the sample expression profile, ***W***_*k*_ ($k=1,2,\cdots ,K$) is an eigensample of column of ***W***, ***V***_*k*_ is an eigenpattern of row of ***V*** ^*T*^, ${{\boldsymbol{V}}}_{j}^{T}$ is the *j*-th column of ***V*** ^*T*^.

To identify the feature genes from ***X***, we should study the critical information of the feature genes. Following the formula, the critical information of feature genes in ***S***_*j*_ can be captured by ***W***_*k*_.

22 $${{\boldsymbol{S}}}_{j}=\sum _{k=1}^{K}{{\boldsymbol{W}}}_{k}{v}_{jk},j=1,2,\cdots n,$$

where ***V*** ^*T*^ contains the coordinates of the *j*-th sample in ***X***. Therefore, the feature genes in ***X*** can be identified by optimizing ***W***.

With ***W*** being processed by OMRFE method, we can get an optimal $\tilde{{\boldsymbol{W}}}$as

23 $$\mathop{{\boldsymbol{W}}}\limits^{ \sim }=[\begin{array}{cccc}{\mathop{w}\limits^{ \sim }}_{11} & {\mathop{w}\limits^{ \sim }}_{12} & \cdots  & {\mathop{w}\limits^{ \sim }}_{1K}\\ {\mathop{w}\limits^{ \sim }}_{21} & {\mathop{w}\limits^{ \sim }}_{22} & \cdots  & {\mathop{w}\limits^{ \sim }}_{2K}\\ \vdots  & \vdots  & \ddots  & \vdots \\ {\mathop{w}\limits^{ \sim }}_{m1} & {\mathop{w}\limits^{ \sim }}_{m2} & \cdots  & {\mathop{w}\limits^{ \sim }}_{mK}\end{array}].$$

Relying on^25^, the feature genes are usually grouped into up-regulated and down-regulated, which are reflected by the positive or negative elements respectively in $\tilde{{\boldsymbol{W}}}$. In this paper, only the absolute value of the elements in $\tilde{{\boldsymbol{W}}}$is considered to identify feature genes. So we sum the elements by rows to obtain the evaluating vector^13^:

24 $$\hat{{\boldsymbol{W}}}={[\begin{array}{cccc}\sum _{k=1}^{K}|{\mathop{w}\limits^{ \sim }}_{1k}| & \sum _{k=1}^{K}|{\mathop{w}\limits^{ \sim }}_{2k}| & \cdots  & \sum _{k=1}^{K}|{\mathop{w}\limits^{ \sim }}_{mk}|\end{array}]}^{T}.$$

Generally, the more differentially expressed the gene is, the larger the corresponding element in $\hat{{\boldsymbol{W}}}$ is. Hence, we can sort the items of $\hat{{\boldsymbol{W}}}$ in a descending order, then take the top *h* (*h* < *m* is a number that can be selected according to the requirement) genes as features.

### Definition of OMBRFE

Based on the TCGA colorectal cancer data, Lee *et al*. integrated the multiple classes of available genomic data to generate the integrated data which included copy number alterations, somatic mutations, methylation and gene expression changes^9^. We can identify the feature genes associated with advanced colorectal cancer in clinical stage via the integrated data. Since different genomic data sets have different peculiarities and distribution, it is inappropriate to treat them as a single data for conventional methods. Different genomic data should have different constraint parameter, so the block ideology is suitable to deal with the integrated data. Therefore, based on OMRFE method, we propose another feature extraction method for the integrated colorectal cancer data named OMBRFE.

Suppose ***X***_*i*_, where $i=1,2,\cdots ,c$, is the *i*-th block of the data matrix ***X*** and *c* is the number of the blocks, the definition of OMBRFE is as follows:

25 $$\mathop{\min }\limits_{{{\boldsymbol{W}}}_{i},{{\boldsymbol{b}}}_{i},{{\boldsymbol{W}}}_{i}^{T}{{\boldsymbol{W}}}_{i}={\boldsymbol{I}}}{\Vert ({{\boldsymbol{X}}}_{i}-{{\boldsymbol{b}}}_{i}{{\boldsymbol{a}}}_{i}^{T}){{\boldsymbol{V}}}_{i}-{{\boldsymbol{W}}}_{i}\Vert }_{2,1}+{\lambda }_{i}{\Vert {{\boldsymbol{W}}}_{{\boldsymbol{i}}}\Vert }_{\ast },$$

where *λ*_*i*_ is the regularization parameter corresponding to ***X***_*i*_. Similar to OMRFE, Eq. (25) can also be solved by the ALM method. Following the ALM method, the optimized ***W***_*i*_ can be obtained. Finally, the optimized integrated ***W*** by integrating *c* optimized block matrices

26 $${\boldsymbol{W}}=[{{\boldsymbol{W}}}_{1},{{\boldsymbol{W}}}_{2},\cdots ,{{\boldsymbol{W}}}_{c}]$$

### Identify feature genes using OMBRFE on colorectal cancer integrated data

**Figure 2:**
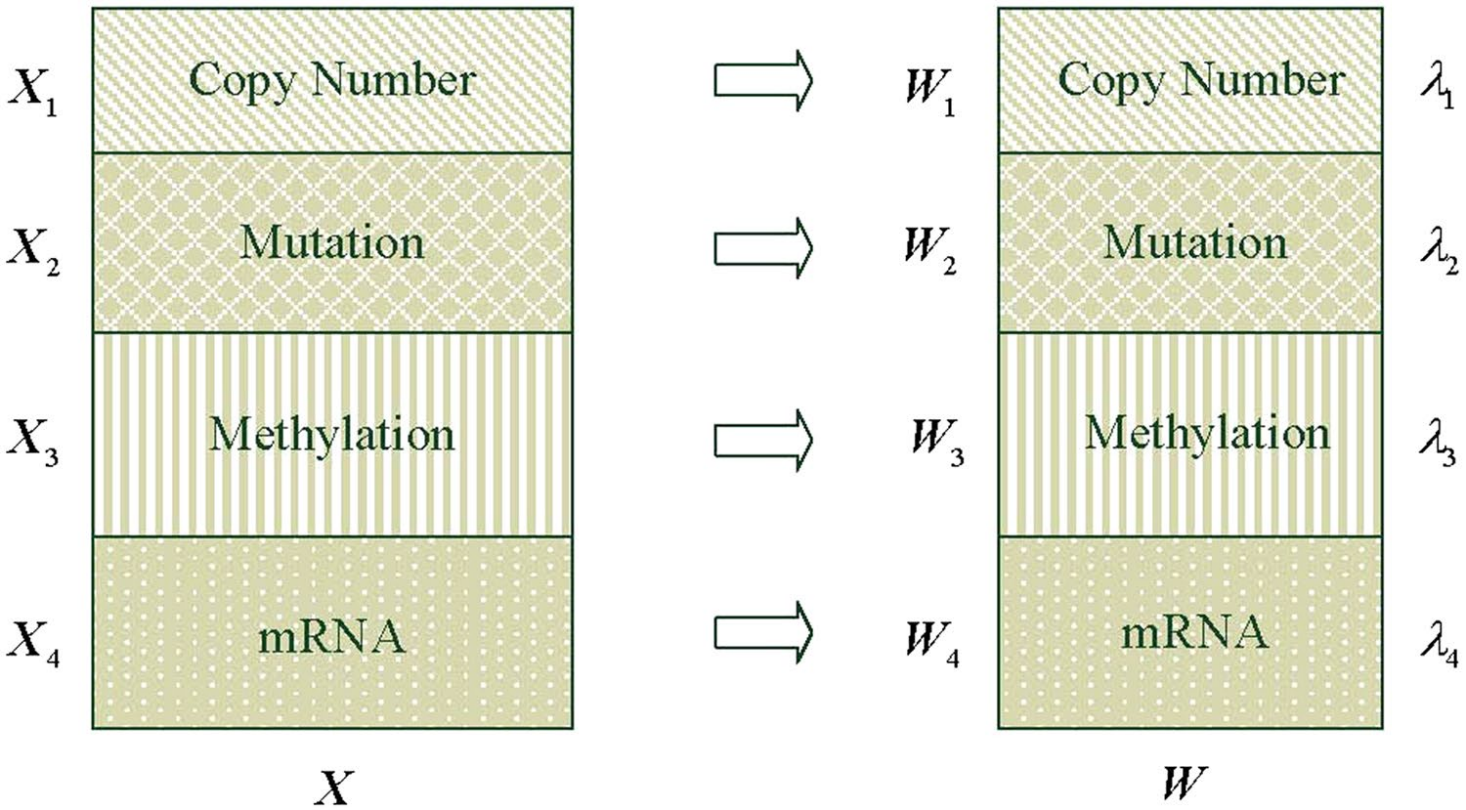
The OMBRFE model for cancer gene identification from colorectal cancer integrated data.

The colorectal cancer integrated data includes copy number alterations, somatic mutations, methylation and mRNA. We can identify the feature genes associated with advanced colorectal cancer in clinical stage via the integrated data. Following OMBRFE, the integrated data should be processed in blocks. So the OMBRFE model for cancer gene identification from colorectal cancer integrated data can be described in Fig. 2.

In Fig. 2, ***X*** is denoted as colorectal cancer integrated data. ***X***_1_, ***X***_2_, ***X***_3_ and ***X***_4_ are the different blocks and denoted as copy number, mutation, methylation and mRNA in the integrated data respectively. According to the OMRFE method, the data matrix ***X*** can be processed to obtain the optimal ***W*** by using the regularization parameter *λ*. Similarly, by using OMBRFE, the blocks ***X***_1_, ***X***_2_, ***X***_3_ and ***X***_4_ can be processed to obtain the optimal ***W***_1_, ***W***_2_, ***W***_3_ and ***W***_4_ via different *λ*_1_, *λ*_2_, *λ*_3_ and *λ*_4_ respectively. Then the optimal ***W*** is denoted as ***W*** = [***W***_1_, ***W***_2_, ***W***_3_, ***W***_4_].

In the primary optimized ***W***, one gene can appear many times when all the four types of genomic data support the gene. That is, one gene has multiple genomic features in the integrated data. And each genomic feature in ***W*** has a score after processed by OMBRFE. Therefore, the score of a gene will be obtained by summing the scores of the same genomic feature in ***W***. Finally, the scores of genes are sorted in descending order and the top *h* (*h* < *m* is a number that can be selected according to the requirement) genes are selected as the feature ones associated with advanced colorectal cancer in clinical stage.

## Results and Discussion

This section shows the experimental results. Firstly, the regularization parameters *λ* in OMRFE and OMBRFE are determined by using synthetic data. Then the effectiveness of the optimal mean and *L*_2,1_-norm in OMRFE and OMBRFE are verified by simulation. Finally, to demonstrate the effectiveness of OMRFE and OMBRFE methods for identifying the feature genes associated with advanced colorectal cancer in clinical stage, PMD^14^, SPCA^26^, RPCA^13^, and CRPCA-OM^16^ are used for comparison.

### Results on synthetic data

For OMRFE and OMBRFE methods, the regularization parameters *λ* need to be determined appropriately. In conventional methods, the value of *λ* is usually given as: $\lambda =\,\max \,{(m,n)}^{1/2}$, where the size of data matrix *X* is *m* × *n*. In our methods, the parameter *λ* needs to be studied. So we define $\lambda ={(l\ast \max (m,n))}^{1/2}$, where the parameter *l* is a constant value. In this paper, the synthetic data is adopted to determine the optimal *λ*.

The synthetic data are generated as ***X*** ~ (0, ∑_4_) with *m* = 5000, *n* = 200. Let ***v***_1_ ~ ***v***_4_ be four 5000-dimensional vectors, such as ${v}_{1k}=1,k=1,\cdots ,125$, and ${v}_{1k}=0,k=126,\cdots ,5000$; *v*_2*k*_ = 1, *k* = 126, $\cdots$, 250, and ${v}_{2k}=0,k\ne 126,\cdots ,250$; ${v}_{3k}=1,k=251,\cdots ,375$, and ${v}_{3k}=0,k\ne 251,\cdots ,375$; ${v}_{4k}=1,k=376,\cdots ,500$, and ${v}_{4k}=0,k\ne 376,\cdots ,500$. Let ***E*** ~ *N*(0, 1) be a noise matrix with 5000-dimension, which is added to ***v***. The four eigenvectors of ∑_4_ can be denoted as ${\tilde{{\boldsymbol{v}}}}_{k}={{\boldsymbol{v}}}_{k}/\Vert {{\boldsymbol{v}}}_{k}\Vert ,k=1,2,3,4$. To make the four eigenvectors dominate, the eigenvalues in ***X*** can be represented as *c*_1_ = 200, *c*_2_ = 150, *c*_3_ = 100, *c*_4_ = 50 and *c*_*k*_ = 1 for $k=5,\cdots ,5000$. The detailed synthetic idea can be found in ^27^ .

**Figure 3:**
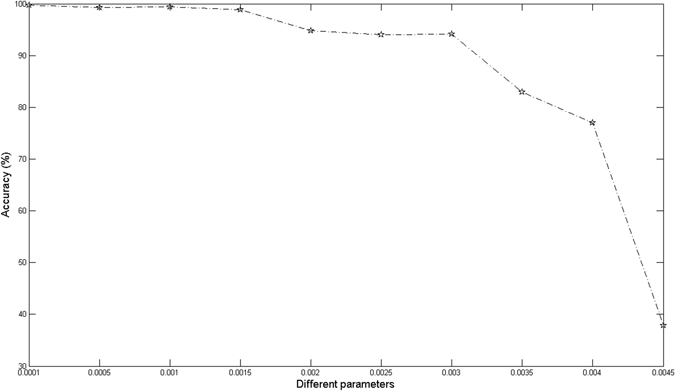
The identification accuracies of OMRFE with different values of *l*.

OMBRFE and OMRFE have the same way in terms of selection of the regularization parameters. For simplicity, we only test the value of *l* in OMRFE. In order to evaluate the performance of different value of *l*, the experiment is repeated for 30 times and the average identification accuracies are reported. For fair comparison, 500 genes are identified by OMRFE. Fig. 3 presents the experimental results of OPMRFE with different values of *l*. From Fig. 3 we can find that the identification accuracies are monotonically decreasing at *l* > 0.001 and the identification accuracies reach the highest point and become stable at *l* ≤ 0.001. Therefore, the regularization parameters in OMRFE can be determined as $\lambda ={(l\ast \max (m,n))}^{1/2},\,(l\le 0.001)$.

In OMBRFE method, we denote the integrated data as ***X***, then the blocks can be defined as ${{\boldsymbol{X}}}_{1}\in {{\mathbb{R}}}^{{m}_{1}\times {n}_{1}}$, ${{\boldsymbol{X}}}_{2}\in {{\mathbb{R}}}^{{m}_{2}\times {n}_{2}}$, ${{\boldsymbol{X}}}_{3}\in {{\mathbb{R}}}^{{m}_{3}\times {n}_{3}}$, ${{\boldsymbol{X}}}_{4}\in {{\mathbb{R}}}^{{m}_{4}\times {n}_{4}}$. Corresponding to the four blocks, the four regularization parameters are denoted as ${\lambda }_{1}={(l\ast \max ({m}_{1},{n}_{1}))}^{1/2},(l\le 0.001)$_,_ ${\lambda }_{2}={(l\ast \max ({m}_{2},{n}_{2}))}^{1/2},(l\le 0.001)$,λ_3_ =(1 * max(*m*_3_,*n*_3_))^1/2^ $(l\le 0.001)$ ${\lambda }_{4}={(l\ast \max ({m}_{4},{n}_{4}))}^{1/2},(l\le 0.001)$. In this paper, the value of *l* is selected as 0.0001 in both OMRFE and OMBRFE.

OMBRFE and OMRFE are robust feature extraction methods with an optimal mean removed. Therefore, how the robustness and optimal mean work in OMRFE and OMBRFE should be studied. Since the two methods are identical in the terms of robustness and optimal mean, for simplicity, only the OMRFE method is validated in this subsection.

**Figure 4:**
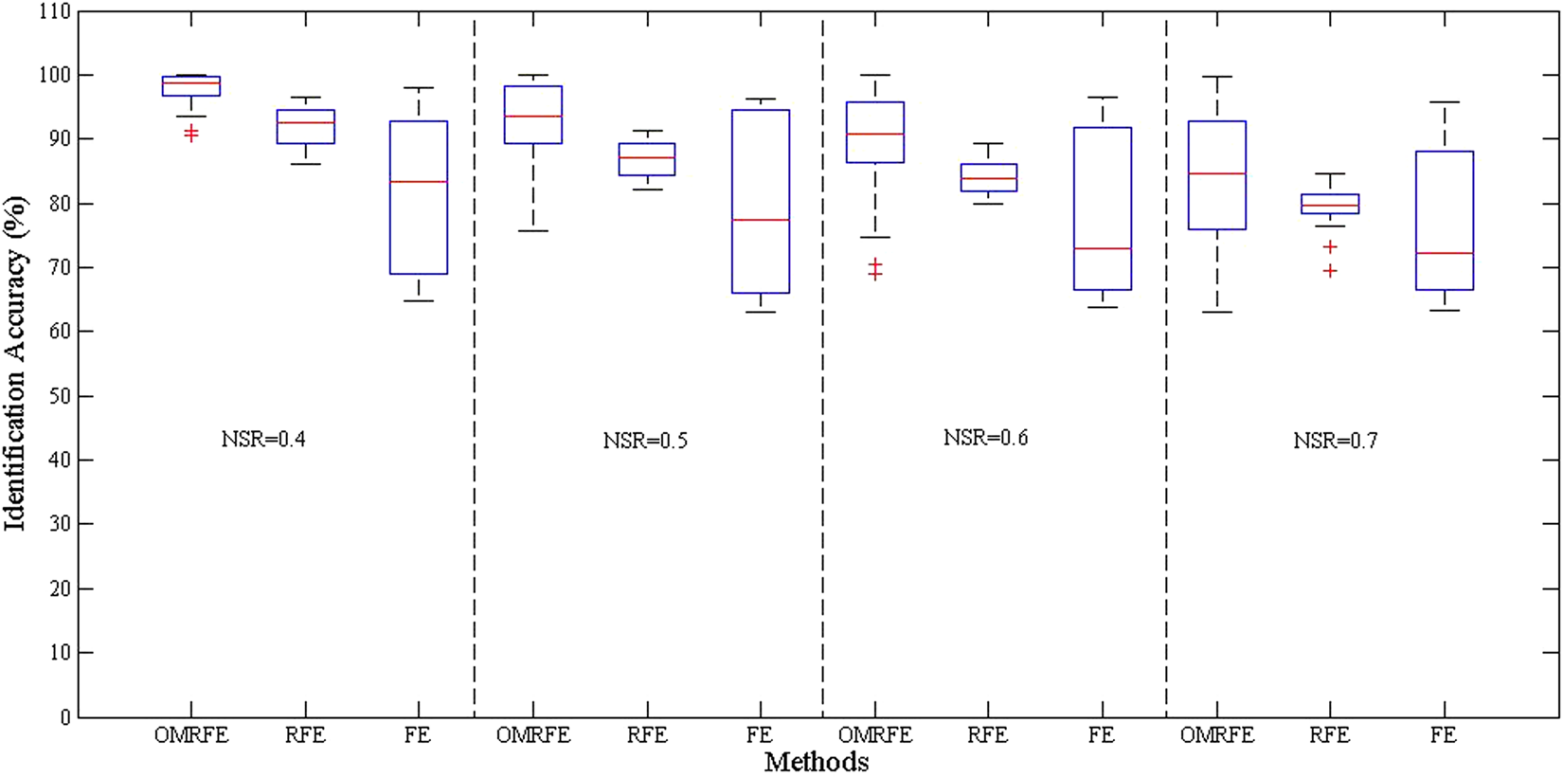
The identification accuracies of OMRFE, RFE and FE, where FE is the feature extraction method with *L*_2_-norm, RFE is the robust feature extraction method with *L*_2,1_-norm, and OMRFE is the robust feature extraction method with *L*_2,1_-norm and an optimal mean removed. NSR is denoted as the noise-to-signal ratio.

We denote FE as the feature extraction method with *L*_2_-norm, RFE the robust feature extraction method with *L*_2,1_-norm, and OMRFE the robust feature extraction method with *L*_2,1_-norm and an optimal mean removed. So the robustness of *L*_2,1_-norm and the optimal mean can be studied by using FE, RFE and OMRFE methods. In this section, we also adopt the synthetic data. In our experiments, different NSR (noise-to-signal ratio) is added to the synthetic data to test the robustness of *L*_2,1_-norm. For fair comparison, the experiments of the three methods are repeated for 30 times respectively, and the results are summarized in Fig. 4. From Fig. 4 we can find that in terms of Inter quartile range (IQR) OMRFE and RFE achieved more robust performance than FE due to the use of *L*_2,1_-norm. And in terms of median identification accuracy, OMRFE achieved higher performance than RFE and FE by removing an optimal mean. It is worth mentioned that, compared with RFE and FE, the extraction performance can be improved in OMRFE by using optimal mean and *L*_2,1_-norm.

### Results on colorectal cancer integrated data

To demonstrate the effectiveness of OMRFE and OMBRFE methods for identifying the feature genes associated with advanced colorectal cancer in clinical stage on colorectal cancer integrated data, the PMD^14^, SPCA^26^, RPCA^13^ and CRPCA-OM^16^ are also used to identify the feature genes. The relevance of genes and advanced colorectal cancer is verified in clinical stage.

Clinical stage information can be obtained from the Broad Firehose (http://gdac.broadinstitute.org), which is one of the Genome Data Analysis (GDACs) for TCGA project. The data files from January 2013 analysis/standardization run of colorectal cancer includes four genomics assays for each sample: DNA copy number variation, somatic mutations by whole exome sequencing, DNA methylation and mRNA expression level by microarray/RNASeq. These genomic data sets were integrated into one data matrix for analysis^9^. The colorectal cancer integrated data set can be downloaded from http://genomeportal.stanford.edu/tcga-crc/.

The colorectal cancer integrated data set consists of 197 samples and 5188 genomic features which integrated copy number alterations, somatic mutations, DNA methylation and mRNA expression. It may have at least one genomic feature for each gene. Among the 5188 genomic features, 1~1117 are copy number, 1118~2030 are somatic mutations, 2031~4108 are DNA methylation and 4109~5188 are mRNA expression.

For fair comparison, 300 genes are identified by PMD, SPCA, RPCA, OMRFE and OMBRFE methods. All 300 genes identified by different methods are listed in supplementary (see Supplementary Material). The GO (Gene Ontology) enrichment of functional annotation of the identified feature genes by the five methods is detected by ToppFun which can be used to describe feature genes in the input or query set and to help discover what functions the feature genes may have in common^28^. The ToppFun tool can be publicly available at http://toppgene.cchmc.org/enrichment.jsp. ToppFun can be used for gene list functional enrichment analysis. It uses as many as 14 annotation categories including GO terms, pathways, protein–protein interactions, protein functional domains, transcription factorbinding sites, microRNAs, gene tissue expressions and literatures. Hypergeometric distribution with Bonferroni correction is used as the standard method for determining statistical significance. Hypergeometric distribution is a standard approach for enrichment analysis. For example, a tool, GOrilla, was presented for discovery and visualization of enriched GO terms by Eden *et al*., and it performs enrichment analysis through hypergeometric distribution^29^. The functional enrichment analysis for pathway, disease, and other functional annotations were conducted using hypergeometric distribution by Zhao *et al*.^30^. Zhou *et al*. presented EasyGO, a web server to perform Gene Ontology Functional enrichment analysis which is done by using hypergeometric test and other two statistical test methods^31^.

The functional enrichment analysis in this study for GO: Biological Process for each gene set was conducted using ToppFun. In this enrichment analysis, all of the human protein-coding genes were used as a background to calculate statistical significance using a hypergeometric model. The Bonferroni correction is also used to correct P-values for enriched annotations based on the hypergeometric test using ToppFun. Finally, the enriched annotations with corrected P-values < 0.01 were identified as overrepresentative annotations for each gene set. The resulting Gene Ontology enrichment results were shown in Table 1.

**Table 1:** The top 10 GO terms corresponding to genes identified by different methods.

Rank	Name	OMBRFE	OMRFE	CRPCA-OM	RPCA	SPCA	PMD	Genes in Genome
		Input	Input	Input	Input	Input	Input	
		PV	PV	PV	PV	PV	PV	
1	Tissue development	**89**	74	72	74	63	74	1794
		**1.07E-23**	1.19E-15	8.85E-14	2.67E-15	8.84E-12	7.13E-15	
2	Cell development	**91**	76	69	75	66	None	1970
		**4.24E-22**	1.59E-14	1.74E-10	1.10E-13	1.97E-11		
3	Regulation of developmental process	**89**	77	71	78	75	72	1912
		**8.63E-22**	9.70E-16	5.84E-12	6.73E-16	1.13E-16	1.69E-12	
4	Regulation of multicellular organismal development	**77**	74	60	73	63	60	1469
		**1.04E-21**	1.74E-20	8.39E-12	1.75E-19	1.04E-15	7.23E-12	
5	Positive regulation of gene expression	**72**	68	60	65	59	66	1332
		**5.44E-21**	4.59E-19	1.31E-13	6.85E-17	2.52E-15	4.38E-17	
6	Positive regulation of nucleobase-containing compound metabolic process	**75**	66	61	64	59	66	1448
		**8.24E-21**	5.28E-16	1.45E-12	1.42E-14	9.94E-14	2.71E-15	
7	Regulation of cell differentiation	**73**	62	61	65	64	57	1405
		**2.63E-20**	2.22E-14	3.94E-13	9.37E-16	3.04E-17	3.55E-11	
8	Positive regulation of nitrogen compound metabolic process	**75**	66	63	64	61	66	1484
		**3.47E-20**	1.76E-15	4.03E-13	4.45E-14	2.28E-14	2.52E-15	
9	Positive regulation of transcription, DNA-templated	**66**	62	57	60	56	67	1221
		**3.48E-19**	3.11E-17	1.43E-13	1.02E-15	3.45E-15	3.11E-15	
10	Positive regulation of cellular biosynthetic process	**75**	65	66	63	62	65	1547
		**3.85E-19**	4.49E-14	7.59E-14	9.61E-13	4.17E-14	5.57E-15	

Table 1 shows the top 10 closely related GO terms corresponding to the genes identified by different methods. In this table, ‘Genes in Genome’ is the number of genes associated with the GO term in global genome; ‘Input’ is the number of genes associated with the GO term from the 300 input genes; PV is the P-value. In Table 1, different methods have different ‘Input’ and different P-value in each GO term. For instance, for the GO term: tissue development, the total number of genes in genome is 1794. Among 300 genes identified by OMBRFE, 89 genes are overlapped with these 1794 genes. The P-value of the 89 genes is calculated by the ToppFun tool.

From Table 1 we can find that the OMRFE method shows better performance than PMD, SPCA, RPCA and CRPCA-OM in majority of results. Comparing OMRFE with CRPCA-OM, only in the term: positive regulation of cellular biosynthetic process, CRPCA-OM method can identify more genes than OMRFE method, but OMRFE has a lower P-value than CRPCA-OM. And OMRFE method can identify more genes and lower P-value than CRPCA-OM in the other 9 GO terms. Comparing OMRFE with RPCA, OMRFE can identify more genes and have lower P-value than RPCA in 7 GO terms except in the following two terms: regulation of developmental process and regulation of cell differentiation. In the term: tissue development, OMRFE has the same number of genes with RPCA, but OMRFE has a lower P-value. In addition to the GO term: regulation of cell differentiation, OMRFE outperforms SPCA in the remaining 9 terms. Though in the terms: tissue development, positive regulation of nucleobase-containing compound metabolic process, positive regulation of nitrogen compound metabolic process and positive regulation of cellular biosynthetic process OMRFE can identify the same number of genes with PMD method, OMRFE has the lower P-value. In the GO term: positive regulation of transcription, DNA-templated, PMD can surpass OMRFE method. OMRFE has a better performance than PMD in the remaining five terms. The results demonstrate that the proposed method OMRFE is quite effective in identifying feature genes.

From Table 1 the effectiveness of OMBRFE method can also be verified. In Table 1, OMBRFE method outperforms other methods on both the number of genes and P-value in all the 10 GO terms in addition to the term: positive regulation of transcription, DNA-templated. In the term: positive regulation of transcription, DNA-templated, OMBRFE identifies less number of genes than PMD. However, OMBRFE has the lower P-value than PMD. Therefore, the performance of OMBRFE explains that the block ideology is appropriate to identify feature genes based on the colorectal cancer integrated data.

To further study the relevance between the feature genes identified by OMBRFE and advanced clinical stage colorectal cancer, these genes are analyzed in a meticulous way.

**Figure 5:**
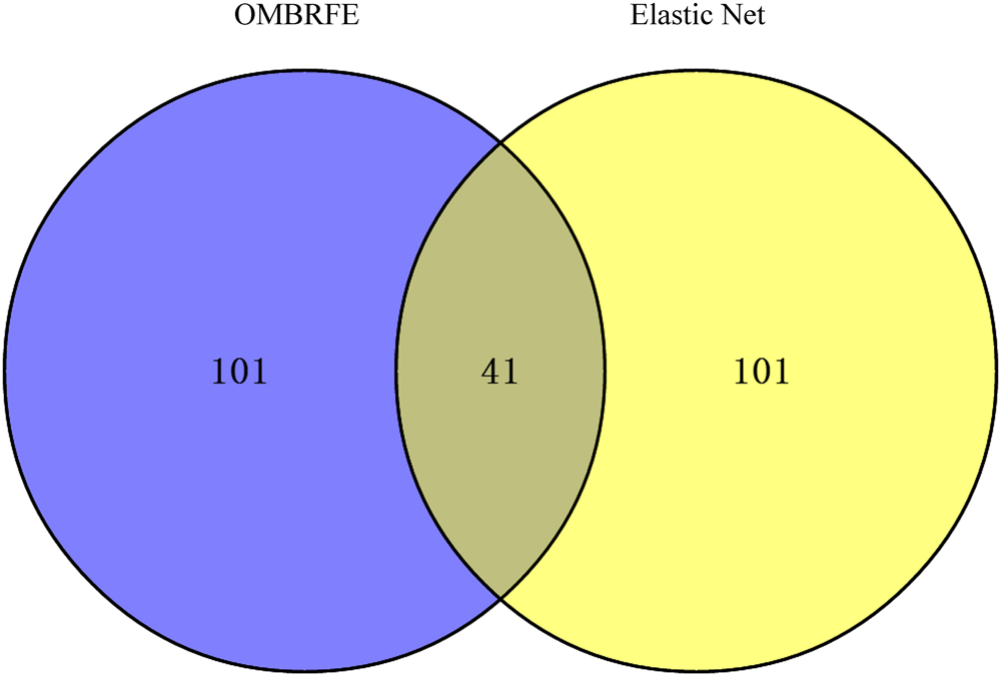
Venn diagram is shown for the feature genes identified by OMBRFE and Elastic Net.

As studied in [9], 142 genes identified by Elastic Net algorithm with integrated analysis delineated advanced clinical stage colorectal cancer. To verify whether the feature genes identified by OMBRFE are associated with the advanced clinical stage colorectal cancer or not, the top 142 feature genes identified by OMBRFE method are selected to make a comparison with the 142 genes identified by Elastic Net algorithm^9^. Fig. 5 shows the Venn diagram for the feature genes identified by both methods. In Fig. 5, 101 genes are OMBRFE and Elastic Net unique respectively. And there are 41 genes overlapped by OMBRFE and Elastic Net. Table 2 summarized the top 20 genes of OMBRFE unique, Elastic Net unique and the overlapping portions of OMBRFE and Elastic Net. In Table 2, the genes identified by OMBRFE unique but neglected by Elastic Net are closely related with colorectal cancer, such as APC and KRAS, which are well known to play an important role in colorectal cancer development since they have a high frequency of genetic aberrations in colorectal cancer^7^. The detailed analysis of feature genes identified by OMBRFE is given in the following.

**Table 2:** The top 20 genes of OMBRFE unique, Elastic Net unique and the overlapping portions of OMBRFE and Elastic Net.

	OMBRFE unique	Overlap	Elastic Net unique
**Gene Symbol**	APC, RUNX3, MSX1, RB1, NRAS, EDNRB, KRAS, OBSCN, MLH1, CACNA1G, PTEN, GPC6, PDE4D, CARD11, RNF213, CCND1, WBSCR17, SOCS2, CSMD1.	GNAS, WT1, MGMT, DIRAS3, TTN, PKD2L1, JAKMIP1, NTRK1, SEMA3B, WRN, BCL2, PLAGL1, PPP2R2C, DMD, RHD, CCND2, PLEKHA4, PIK3R1, PRDM16, FCRL4.	SYK, DDX5, ADRA2C, HSD17B2, HIST1H4I, FOXP4, REEP5, PDK4, OR51E2, S100P, HIP1, ZNF570, SDHC, DDIT3, CRTC1, SLC22A11, CYP26B1, GPR125, TNFAIP3, CATSPER4.

To further study the function of the feature genes identified by OMBRFE, they are analyzed in a meticulous way. For simplicity, the top 20 genes are taken into consideration.

Firstly, the detailed functions of the 20 genes are introduced in Table 3. From Table 3 we can find that all the 20 identified genes are closely related to cancers. The COSMIC (Catalogue of Somatic Mutation in Cancer) database contains 484 genes that have been shown to be closely related to cancer development and thus are established or candidate cancer genes^7^. Among the 20 extracted genes, 9 genes overlapped with the COSMIC study. They are GNAS, APC, WT1, RB1, NRAS, KRAS, MLH1, PTEN and NTRK1.

**Table 3:** The detailed information of the top 20 genes identified by OMBRFE.

NO.	Gene Symbol	Location	Function of Genes
1	GNAS	20q13.3	It gives rise to maternally, paternally, and biallelically expressed transcripts that are derived from four alternative promoters and 5’ exons. Colloid carcinoma associated with intraductal papillary mucinous neoplasms and its intestinal-type preinvasive precursor are associated with high frequencies of GNAS mutations.
2	APC	5q21-q22	This gene encodes a tumor suppressor protein that acts as an antagonist of the Wnt signaling pathway. It is also involved in other processes including cell migration and adhesion, transcriptional activation, and apoptosis.
3	WT1	11p13	This gene encodes a transcription factor that contains four zinc-finger motifs at the C-terminus and a proline/glutamine-rich DNA-binding domain at the N-terminus. WT1 is a major regulator of tumor angiogenesis and progression.
4	MGMT	10q26	Alkylating agents are potent carcinogens that can result in cell death, mutation and cancer. The protein encoded by this gene is a DNA repair protein that is involved in cellular defense against mutagenesis and toxicity from alkylating agents.
5	RUNX3	1p36	This gene encodes a member of the runt domain-containing family of transcription factors. It functions as a tumor suppressor, and the gene is frequently deleted or transcriptionally silenced in cancer.
6	DIRAS3	1p31	This gene encodes a member of the ras superfamily. This gene is imprinted gene with monoallelic expression of the paternal allele which is associated with growth suppression. The encoded protein may also play a role autophagy in certain cancer cells by regulating the autophagosome initiation complex.
7	MSX1	4p16.2	This gene encodes a member of the muscle segment homeobox gene family. The encoded protein functions as a transcriptional repressor during embryogenesis through interactions with components of the core transcription complex and other homeoproteins.
8	RB1	13q14.2	The protein encoded by this gene is a negative regulator of the cell cycle and was the first tumor suppressor gene found. The encoded protein also stabilizes constitutive heterochromatin to maintain the overall chromatin structure.
9	TTN	2q31	This gene encodes a large abundant protein of striated muscle. The product of this gene is divided into two regions, a N-terminal I-band and a C-terminal A-band. DNA sequence analysis of patients with dilated cardiomyopathy shows that genetic variation in TTN gene contributes to a 14% of the cases.
10	NRAS	1p13.2	This is an N-ras oncogene encoding a membrane protein that shuttles between the Golgi apparatus and the plasma membrane. Mutations in this gene have been associated with somatic rectal cancer, follicular thyroid cancer, autoimmune lymphoproliferative syndrome, Noonan syndrome, and juvenile myelomonocytic leukemia.
11	EDNRB	13q22	The protein encoded by this gene is a G protein-coupled receptor which activates a phosphatidylinositol-calcium second messenger system. Its ligand, endothelin, consists of a family of three potent vasoactive peptides: ET1, ET2, and ET3. Studies suggest that the multigenic disorder, Hirschsprung disease type 2, is due to mutations in the endothelin receptor type B gene.
12	KRAS	12p12.1	This gene, a Kirsten ras oncogene homolog from the mammalian ras gene family, encodes a protein that is a member of the small GTPase superfamily. The transforming protein that results is implicated in various malignancies, including lung adenocarcinoma, mucinous adenoma, ductal carcinoma of the pancreas and colorectal carcinoma.
13	OBSCN	1q42.13	The obscurin gene spans more than 150 kb, contains over 80 exons and encodes a protein of approximately 720 kDa. The encoded protein contains 68 Ig domains, 2 fibronectin domains, 1 calcium/calmodulin-binding domain, 1 RhoGEF domain with an associated PH domain, and 2 serine-threonine kinase domains.
14	PKD2L1	10q24	This gene encodes a member of the polycystin protein family. The encoded protein contains multiple transmembrane domains, and cytoplasmic N- and C-termini. The protein may be an integral membrane protein involved in cell-cell/matrix interactions.
15	MLH1	3p21.3	This gene was identified as a locus frequently mutated in hereditary nonpolyposis colon cancer (HNPCC). It is a human homolog of the E. coli DNA mismatch repair gene mutL, consistent with the characteristic alterations in microsatellite sequences (RER+ phenotype) found in HNPCC.
16	CACNA1G	17q22	Voltage-sensitive calcium channels mediate the entry of calcium ions into excitable cells, and are also involved in a variety of calcium-dependent processes, including muscle contraction, hormone or neurotransmitter release, gene expression, cell motility, cell division, and cell death. This gene encodes a T-type, low-voltage activated calcium channel. The function of T-type channels is important for the proliferation of human ovarian cancer cells.
17	PTEN	10q23.3	This gene was identified as a tumor suppressor that is mutated in a large number of cancers at high frequency. The protein encoded by this gene is a phosphatidylinositol-3,4,5-trisphosphate 3-phosphatase.
18	JAKMIP1	4p16.1	Janus kinase and microtubule interacting protein 1. Overexpression of JAKMIP1 associates with Wnt/β-catenin pathway activation and promotes cancer cell proliferation *in vitro*.
19	NTRK1	1q21-q22	This gene encodes a member of the neurotrophic tyrosine kinase receptor (NTKR) family. The presence of this kinase leads to cell differentiation and may play a role in specifying sensory neuron subtypes. Mutations in this gene have been associated with congenital insensitivity to pain, anhidrosis, self-mutilating behavior, mental retardation and cancer.
20	GPC6	13q32	The glypicans comprise a family of glycosylphosphatidylinositol-anchored heparan sulfate proteoglycans, and they have been implicated in the control of cell growth and cell division. The glypican encoded by this gene is a putative cell surface coreceptor for growth factors, extracellular matrix proteins, proteases and anti-proteases.

To further study whether these genes are associated with advanced colorectal cancer or not, they are verified according to the existing literatures. Depending on [9], 142 genes are proved be associated with advanced colorectal cancer in clinical stage. Among the 20 genes identified by OMBRFE, there are 8 genes overlapped with the 142 genes. The symbols of these 8 genes are GNAS, WT1, MGMT, DIRAS3, TTN, PKD2L1, JAKMTP1 and NTRK1. The remaining 12 genes should be studied to demonstrate the relevance between them and advanced colorectal cancer.

12 genes are verified to be associated with advanced colorectal cancer in clinical stage by existing literatures. The 12 gene symbols are given as follows: APC, KRAS, MSX1, RB1, NRAS, GPC6, EDNRB, OBSCN, MLH1, RUNX3, CACNA1G and PTEN. In later analysis, these genes are marked in bold in order to make them more eye-catching.

In a heavily pretreated patient with advanced colorectal cancer carrying mutations in **APC** and **KRAS** genes, Gamerith *et al*. showed an early metabolic response and enhanced NK cell activity to monotherapy with lenalidomide. After subsequent lenalidomide/cetuximab combination treatment, the patient had progressive disease^32^. In vitro studies using non-colonic cell lines have indicated that miR-148a exerts a tumor suppressive function by targeting several genes such as PXR, TGIF2, **MSX1**, CDC25B, DNMT1 and DNMT3b. The dysregulation of miR-148a has been implicated in colorectal cancer^33^. In [31], 17 patients with locally advanced rectal adenocarcinomas, clinical stage II, III according to IUCC were enrolled into the pilot study of Garajová *et al*. Gene expression data analysis based on SAM (Significance Analysis of Microarrays) and t-test methods identified 8 genes (**RB1**, RBBP4, HYOUI, JUNB, MDM4, CANX, MMP2, TCF7L2) significantly upregulated in nonresponders^34^. According to [32], the absence of an oncogenic **KRAS** or **NRAS** mutation has been found to predict clinical benefit from treatment with anti-EGFR antibodies in colorectal cancer^35^. A group of genes previously reported as the most frequently mutated genes in non-hypermutated colorectal cancer in [33]: TP53, **APC**, **KRAS**, CSMD3, TCF7L2, PI3KCA, FBXW7, SOX9, SMAD4, PTPRD, **GPC6**, **EDNRB**, GNAS, AMER1, **NRAS**, KIAA1804, CTNNB1, ACVR1B, and SMAD2^36^. In [34], 36 genes were found to have the most frequent mutations in colorectal cancer and involved functions/pathways. These genes can well exemplify the reason that in clinical practice both patients and physicians’ expectations with targeted therapy are, so far, largely unmet. Among the 12 genes identified by OMBRFE, there are 5 genes overlapped with these 36 genes: **APC**, **KRAS**, **OBSCN**, **MLH1** and **PTEN** ^37^. In [35], one hundred fifty patients with locally advanced rectal cancer, treated within a phase III clinical trial, were included in this analysis. CIMP was assessed by methylation specific PCR (MSP) using RUNX3, SOCS1, NEUROG1, IGF2, and CACNA1G as a marker panel. **CACNA1G** encodes a T-type calcium channel and its aberrant methylation of CACNA1G was also shown in other cancers. Inactivation of CACNA1G may play a role in cancer development by modulating calcium signaling, which potentially affects cell proliferation and apoptosis. **RUNX3** has a tumor suppressor function and is associated to disease stage and patient outcome in colorectal cancer when expression was decreased by promoter methylation^38^.

By studying these genes and related literatures, we can find that several genes (APC, KRAS and NRAS) appeared multiple times when we analyze other genes. For example, in literature [33], GPC6 and EDNRB are proved to be associated with colorectal cancer, while APC, KRAS and NRAS are also proved. This suggests that APC, KRAS and NRAS, especially APC and KRAS, may be absolutely the cause of colorectal cancer.

To sum up, all the 20 genes identified by using OMBRFE are proved to be closely associated with advanced colorectal cancer in clinical stage. Moreover, the results also demonstrate that our OMBRFE method is quite effective in identifying colorectal cancer genes on colorectal cancer integrated data.

## Conclusions

In this paper, we conducted two feature extraction methods Optimal Mean based Robust Feature Extraction method (OMRFE) and Optimal Mean based Block Robust Feature Extraction method (OMBRFE) to identify the feature genes associated with advanced colorectal cancer in clinical stage by using the integrated colorectal cancer data. Thanks to the optimal mean and *L*_2,1_-norm, OMRFE shows better performance on the integrated data than conventional methods. The OMBRFE introduces the block ideology into OMRFE and imposes different regularization parameters on different genomic feature data in colorectal cancer integrated data. Experimental studies demonstrate that OMBRFE is more effective than previous feature extraction methods (including OMRFE) to identify the feature genes on colorectal cancer integrated data. Furthermore, genes identified by OMBRFE are verified to be closely associated with advanced colorectal cancer in clinical stage.

## Electronic supplementary material

Supplementary Information
