# Supplementary material for: An Optimal Mean Based Block Robust Feature Extraction Method to Identify Colorectal Cancer Genes with Integrated Data
Source: Sci Rep. 2017 Aug 17;7:8584. doi: 10.1038/s41598-017-08881-3 (PMC5561268; doi:10.1038/s41598-017-08881-3)
Supplement: Supplementary file 1 — Supplementary Information [file 41598_2017_8881_MOESM1_ESM.doc]

Supplementary Information

**An Optimal Mean Based Block Robust Feature Extraction Method to Identify Colorectal Cancer Genes with Integrated Data**

Jian Liu^1^, Yuhu Cheng^1^, Xuesong Wang^1^*, Lin Zhang^1^, Hui Liu^1^

^1^ School of Information and Control Engineering, China University of Mining and Technology, Xuzhou, 221116, China.

* Corresponding author

X.S.W. E-mail address: wangxuesongcumt@163.com; Tel: +86-139-1345-5365

All 300 genes identified by different methods are shown as follows.

| **OMBRFE** | **OMRFE** | **CRPCA-OM** | **RPCA** | **SPCA** | **PMD** |
| --- | --- | --- | --- | --- | --- |
| GNAS | ABHD12B | GNAS | ABHD12B | TTL | CREB3L1 |
| APC | REG1B | MGMT | CKMT2 | GMDS | PDZD3 |
| WT1 | BTNL8 | RUNX3 | REG1B | ZMYM2 | GNA11 |
| MGMT | PLA2G12B | WT1 | PLA2G12B | IFT88 | IGSF11 |
| RUNX3 | FABP1 | CCND2 | FABP1 | MTMR6 | GATA1 |
| DIRAS3 | CKMT2 | SEMA3B | GRM8 | ATP8A2 | ARHGAP26 |
| MSX1 | WT1 | BRCA1 | RBP2 | WASF3 | PSMB8 |
| RB1 | GRM8 | RB1 | PLA2G2A | RPL21 | MALL |
| TTN | EDAR | DIRAS3 | WT1 | RASL11A | FOXO1 |
| NRAS | RBP2 | CCND1 | EDAR | GTF3A | KCNG3 |
| EDNRB | MLF1 | MSX1 | BTNL8 | MTIF3 | TOX2 |
| KRAS | SP8 | ERBB2 | CXCL11 | LNX2 | IRF2 |
| OBSCN | ZDHHC11 | BCL2 | MSLN | POLR1D | NAB1 |
| PKD2L1 | PLA2G2A | KRAS | KLHL34 | GSX1 | HOXA11 |
| MLH1 | KLHL34 | SP8 | ZDHHC11 | CDX2 | PKP3 |
| CACNA1G | WIF1 | MLH1 | MLF1 | PDX1 | CKMT2 |
| PTEN | SEC14L4 | TMED6 | SEC14L4 | PRHOXNB | SLC44A4 |
| JAKMIP1 | SOX2 | CKMT2 | ACSL6 | FLT3 | GRB7 |
| NTRK1 | MSLN | DNM2 | HOXA9 | PAN3 | STOX2 |
| GPC6 | COL9A3 | ABHD12B | COL9A3 | FLT1 | SERPIND1 |
| PDE4D | TRIM29 | SOCS1 | TMED6 | BRCA2 | CASP3 |
| SEMA3B | HOXA9 | CES3 | PPP2R2C | LHFP | POLR1D |
| CARD11 | TMED6 | SLC22A18AS | ADRA2C | FOXO1 | TGFBR2 |
| RNF213 | WBSCR27 | PRDM2 | TNNC1 | SLC25A15 | SEC14L4 |
| WRN | ANPEP | KLHL34 | WIF1 | LCP1 | RASL11A |
| BCL2 | ACSL6 | NCOA2 | HOXD13 | RB1 | KCTD12 |
| CCND1 | HSD17B2 | F5 | PDZK1 | NEK3 | TNFAIP8L2 |
| PLAGL1 | PRDM16 | HERPUD1 | HOXD11 | KLF5 | HEYL |
| WBSCR17 | HOXD11 | BTNL8 | HSD17B2 | KCTD12 | MTMR11 |
| SOCS2 | CXCL11 | TGFBR2 | RARRES1 | EDNRB | C1orf172 |
| CSMD1 | ADRA2C | WIF1 | POU2AF1 | GPC6 | CNBP |
| PAX3 | KIT | PTEN | SP8 | IPO5 | TMEM139 |
| CPZ | F5 | CRAMP1L | AMPD1 | ERCC5 | HNMT |
| PPP2R2C | PPP2R2C | CDH1 | GRHL3 | F7 | ALDH2 |
| DMD | HOXA13 | MAG | SOX2 | UPF3A | SLC35D2 |
| ATM | HMGA2 | ZNF683 | PRDM16 | ENOSF1 | ESRRA |
| BCL9L | EPHX4 | GPR56 | WBSCR27 | ROCK1 | SMPD2 |
| ERBB2 | HOXD13 | DPPA2 | HOXA13 | ZNF521 | CCDC6 |
| MYH11 | RARRES1 | EWSR1 | NR0B2 | SS18 | EDAR |
| DCAF4L2 | GPC3 | TFRC | TNFRSF17 | SMAD2 | RCC1 |
| PKHD1 | SULT2A1 | APC | EPHX4 | ME2 | H3F3A |
| CTNNB1 | G0S2 | NF1 | ANPEP | ELAC1 | ATP8A2 |
| RHD | GRHL3 | FHIT | SULT2A1 | SMAD4 | CBLC |
| FFAR2 | CDKN2A | BRAF | PRAP1 | DEFB116 | SOX2 |
| SOX9 | TNNC1 | SHANK1 | TRIM29 | DEFB118 | NBN |
| ABHD12B | PDZK1 | FANCF | ZNF683 | DEFB119 | S100P |
| REG1B | SCG5 | DNMT3A | CATSPERB | DEFB121 | ESRP1 |
| CDH11 | MAEL | PLAGL1 | SCG5 | DEFB123 | FAM46B |
| CCND2 | AMPD1 | NDRG1 | FOXC1 | DEFB124 | PLA2G2A |
| EVC2 | ZNF683 | GNMT | CASP1 | REM1 | KIF5B |
| PDGFRB | NR0B2 | CYTIP | CBFA2T3 | HM13 | ELF3 |
| GPHN | HTR1D | SH3GL1 | KIT | ID1 | CDX2 |
| PLEKHA4 | WASF3 | MSH3 | G0S2 | COX4I2 | PPARG |
| PIK3R1 | PRAP1 | DNAI1 | F5 | BCL2L1 | RPL22 |
| COL1A1 | FOXC1 | VTI1A | CDKN2A | ASXL1 | FBXO17 |
| SMAD2 | CCDC110 | MYCN | CCDC110 | MAP1LC3A | SULT2A1 |
| PRDM16 | MSX1 | PAFAH1B2 | GPD1 | NNAT | C1orf210 |
| BCHE | CARD11 | CDIPT | MYOM3 | CTNNBL1 | CSMD1 |
| CDKN2A | ZNF595 | MLF1 | RASL11A | PPP1R16B | FANCF |
| AMPD1 | MGMT | RASL11A | MYEF2 | MAFB | PML |
| FCRL4 | RANBP17 | SYK | COL1A1 | TOP1 | MYO1A |
| RUNX1 | S100P | REG1B | SORBS2 | MYBL2 | RPL21 |
| WASF3 | POU2AF1 | HOXA9 | MYOM2 | GTSF1L | REEP5 |
| SCUBE2 | MYOM2 | CCDC78 | DAPP1 | TOX2 | ABI1 |
| MSH3 | MYOM3 | CYLD | GBP4 | JPH2 | PPP2R1A |
| EGFR | MUC1 | PDZK1 | HTR1D | C20orf111 | SMPDL3B |
| LIFR | SORBS2 | MN1 | GPC3 | GDAP1L1 | PDZK1 |
| TCF7L2 | SULT1C4 | COL5A2 | MGMT | R3HDML | TCEA3 |
| TET1 | CATSPERB | SERPIND1 | ZNF595 | HNF4A | ETV6 |
| CXCR7 | DRD5 | SH3BGRL3 | MUC1 | SERINC3 | MYOM3 |
| BRCA1 | DOK7 | TMEM139 | ID1 | PKIG | ZNF683 |
| CAMTA1 | CBFA2T3 | CBLC | SFTA2 | ADA | CATSPERB |
| HOXA9 | PEX6 | GRM8 | FAP | WISP2 | NTRK1 |
| CRMP1 | MYEF2 | DNASE1L2 | S100P | SDC4 | BCHE |
| GPC3 | CASP1 | FFAR2 | MS4A1 | MC3R | CIITA |
| MLF1 | SFTA2 | JAKMIP1 | MSX1 | SPO11 | SORBS2 |
| PGC | RET | FLCN | FCGR2B | GNAS | NDUFA13 |
| NF1 | FZD3 | NFKB2 | CARD11 | CTSZ | MTMR6 |
| TNXB | CCND2 | PPP1R14D | ANKRD37 | SS18L1 | RPN1 |
| ROS1 | ID1 | EFNA1 | SLC30A2 | COL9A3 | HPS1 |
| RET | GPD1 | CLTCL1 | HMGA2 | CREB3L1 | SDHD |
| ZNF521 | RASL11A | GPC6 | IFI6 | PDZD3 | TRIM63 |
| MYOM2 | TNFRSF17 | PBRM1 | WASF3 | UPF3A | MYC |
| ANPEP | ANKRD37 | RECQL4 | CA3 | GFI1 | GBP4 |
| SLA | TP53 | SERGEF | CCND2 | KLHL6 | KLF5 |
| KAT6B | CES3 | ITK | APOD | WNT10B | IFT88 |
| PTK7 | GGTLC1 | TSC1 | DRD5 | SLC25A15 | GPC3 |
| CIITA | IFI6 | TSC22D4 | GZMB | ACSL1 | RAP1GDS1 |
| CYTL1 | NKX2-1 | MLF1IP | GGTLC1 | IPO5 | FCGR2B |
| DNAH9 | DAPP1 | GRK4 | FZD3 | TOX2 | RHD |
| NTRK3 | APOD | CASP3 | DOK7 | NAB1 | PAQR6 |
| MYEF2 | AMT | CHEK2 | PPP1R14D | NONO | FAS |
| PPP2R2B | TAF15 | RHOH | MED18 | DMD | MRPS18A |
| MYBL2 | XDH | HOXD13 | RET | PDCD1LG2 | EFNA1 |
| ABL2 | TRIM63 | PPP2R1A | RANBP17 | CKMT2 | REG1B |
| USP6 | PLAG1 | PAX3 | PLAG1 | ANKRD37 | MYH11 |
| TSHR | MS4A1 | ELF4 | CD74 | POU2AF1 | DDAH2 |
| UBR5 | WWTR1 | SOX2 | XDH | GGTLC1 | LASP1 |
| NLGN3 | SYTL1 | FAM78A | TRIM22 | GRHL3 | FOXP4 |
| GLIPR1L2 | MED18 | SMARCA4 | SYTL1 | DIRAS3 | CDK12 |
| FLT1 | NAT8L | HSP90AA1 | PEX6 | ZDHHC11 | CD79A |
| SCN5A | SLC30A2 | DAPP1 | AMT | SEC14L4 | ERBB3 |
| FANCA | RNF212 | FLVCR2 | TCEA3 | AMT | SYK |
| GRIK3 | TUBB2B | GRB7 | PTK7 | KCTD12 | HNF4A |
| HOXC11 | NPDC1 | BAP1 | TAF15 | TNFAIP8L2 | TNFRSF11A |
| SERPIND1 | CA3 | ETV5 | AGBL2 | MTMR11 | CEBPA |
| CD79B | RUNX3 | AMDHD2 | TRIM63 | IL21R | IL7R |
| HMGCL | RHOD | C2orf44 | PKD2L1 | PDE4D | JAK1 |
| HOXD11 | PTPN7 | ACOX3 | CES3 | COL9A3 | JUN |
| TRIM63 | PKD2L1 | ZBTB20 | KCTD12 | NGEF | MITF |
| MAP2K4 | COL1A1 | CCDC80 | HOXA11 | ZFYVE28 | HOXA13 |
| SHANK1 | KCTD12 | IFI6 | GBP1 | F5 | C6orf223 |
| TP53 | NR4A3 | KTN1 | ETV5 | LCP1 | PPP1R1B |
| PRDM12 | NFIB | HMGA2 | ARHGEF10 | ASXL1 | ZNF593 |
| WIF1 | TM4SF1 | GMEB1 | FGFR2 | SOX2 | SFTA2 |
| HAS1 | ACMSD | MYOD1 | RHOD | S100P | GATA3 |
| SMARCA4 | ZBTB16 | CDK12 | KBTBD11 | PLA2G2A | C11orf52 |
| CUX1 | HOXC11 | IKZF1 | SLC39A5 | PPARG | CES3 |
| GZMB | FAP | VHL | GFI1 | LCK | GBP1 |
| MS4A1 | TCEA3 | UBR5 | CTSZ | FGFR2 | DNM2 |
| BCOR | HOXC13 | AKAP9 | NAT8L | PTK7 | HTR1D |
| AFAP1 | TRIM22 | RAP1GDS1 | TUBB2B | SULT2A1 | SBDS |
| NR0B2 | ALK | HOXD3 | TP53 | PTCH1 | SLC39A8 |
| EXT1 | ARHGEF10 | CARD11 | NPDC1 | FANCF | MAP1LC3A |
| ALK | SERPIND1 | MAP3K6 | SPON2 | PML | SH3BGRL3 |
| ITK | GBP4 | POLN | CYTIP | FAP | RPS6KA1 |
| RNF113A | PDK4 | CHIC2 | RUNX3 | MYO1A | TAF15 |
| GRM8 | AGBL2 | SUSD1 | BCHE | ETV5 | HAMP |
| CES3 | GGTLC1 | ROCK1 | CYTL1 | PDZK1 | ELMO3 |
| CBLB | PTK7 | XPC | MTMR11 | SS18L1 | IL18R1 |
| BTNL8 | ROS1 | FBXO11 | FGFR3 | CYTIP | BCL10 |
| DRP2 | FGFR2 | CARS | FAS | MYOM3 | OLIG2 |
| FHIT | HOXC12 | MOSPD3 | WFS1 | ZNF683 | MYO1B |
| PRDM2 | GTF3A | HSD3B7 | JAKMIP1 | CATSPERB | TRIM22 |
| CKMT2 | CRIP3 | KLF14 | ABLIM2 | BCL2 | SLC44A2 |
| ELF3 | BCL11A | HOXC11 | ZNF331 | F7 | BTG1 |
| KLHL34 | HOXA11 | CA3 | SMO | RHOH | USP49 |
| DNAI1 | FOXL2 | TACC3 | AQP1 | DAPP1 | MUC1 |
| 6-Sep | CCDC78 | SOCS2 | BIRC3 | CIITA | IDH2 |
| ERCC5 | FGFR3 | AMPD1 | BCL2 | TRIM63 | CXCL11 |
| GAS7 | SMO | NTRK1 | C6orf223 | WIF1 | CHCHD7 |
| RUNX1T1 | ABLIM2 | SERINC3 | PDZD3 | MYC | LRIG3 |
| EXTL1 | SLC16A7 | EDNRB | PAQR6 | GBP4 | KBTBD11 |
| BRIP1 | IL7R | TUBB2B | ROS1 | FZD3 | KDR |
| DLGAP2 | SLC39A5 | CASP1 | KCNG3 | TMED6 | TMEM45B |
| ELF4 | ZNF331 | EGFR | PPARG | RAB42 | CRYAA |
| IL26 | LCK | XPO5 | MYO1A | CTNNBL1 | SP100 |
| RPE65 | PAQR6 | DAK | TRERF1 | FCGR2B | STX12 |
| GRIP1 | KBTBD11 | PLEKHA4 | CDH11 | SLC16A7 | CDH1 |
| LNX2 | FCGR2B | PPP2R3A | BCL11A | FAS | CD52 |
| PDCD1LG2 | GZMB | CTSZ | CIITA | REG1B | HOXA9 |
| SH3TC1 | CYP26B1 | FOXP4 | JUN | DDAH2 | LNX2 |
| EIF4A2 | BCL2 | XPA | PDK4 | WASF3 | PTAFR |
| ACSL6 | ECT2L | CYB561D1 | F7 | PRKAR2B | ABP1 |
| GMEB1 | FAS | MUC1 | TFRC | HNF4A | FCN3 |
| ACVRL1 | AQP1 | ELAC1 | PLEKHA4 | TNFRSF11A | FOXP1 |
| SMAD4 | KCNG3 | BCL7A | CD52 | CEBPA | PKD2L1 |
| DNMT3A | PLAGL1 | SRF | OMD | IL7R | XDH |
| HLF | ETV5 | ZDHHC11 | RNF212 | PLEKHA4 | COL1A1 |
| DICER1 | DNASE1L2 | SMO | HOXC11 | CDKN2A | NAT8L |
| FAM65A | SPO11 | MTMR11 | WHSC1L1 | JUN | TPM3 |
| SP8 | TNFRSF11A | MAMDC4 | ACSL1 | SCG5 | SLC39A5 |
| ZNF572 | IL18R1 | TP53 | MYH11 | HOXD11 | IL18BP |
| GATA3 | MYO1A | MIER3 | CCDC78 | SMO | PDGFRB |
| ARHGEF10 | ATP6V0D2 | SP100 | NDRG1 | FGFR3 | CASP1 |
| ZNF570 | GFI1 | MYBL2 | EVI2A | PBX1 | SLC30A2 |
| CRAMP1L | KAT6A | KDM5A | FGFR1 | SYTL1 | DPPA2 |
| SLC16A7 | SLC39A8 | PPP2R2C | TMEM176A | FAM78A | TUBB2B |
| MSN | PRDM12 | CREB3L1 | TM4SF1 | CD79B | AFAP1 |
| PAX7 | CYTL1 | MYD88 | SULT1C4 | ZNF514 | ERBB2 |
| LTV1 | PPP1R14D | RPA2 | NFIB | BCL6 | HEY1 |
| VTI1A | PRKAR2B | GATA1 | LCK | SFTA2 | CANT1 |
| FAP | IL22RA1 | PPP1R16B | PRDM12 | FLT1 | RARRES1 |
| SETD2 | PAX3 | PAQR7 | ADA | SMAD2 | PIK3R1 |
| DRD5 | ZNF572 | PEX6 | ATP6V0D2 | GBP1 | MLLT4 |
| MAN1C1 | PLEKHA4 | MED12 | COL5A2 | NR0B2 | LPP |
| MAEL | NDRG1 | CDKN2A | PLAGL1 | HTR1D | HOXC13 |
| IQCK | OMD | CSMD1 | RUNX1T1 | SLC39A8 | SERINC3 |
| COL5A2 | TRERF1 | CD79B | ZFYVE28 | FFAR2 | PLAGL1 |
| ZNF683 | CYTIP | FLI1 | SLC16A7 | MAP1LC3A | KLF6 |
| TMEM176A | ETV1 | SDHAF2 | IL7R | IL18R1 | ELN |
| SORBS2 | JUN | FGFR1OP | ZNF572 | ADRA2C | SFN |
| CNTRL | NGEF | CBLB | MDFI | TRIM22 | ABLIM2 |
| TCTE1 | CD74 | STK32B | GTF3A | MUC1 | TMEM63B |
| HTR1D | C6orf223 | PKD2L1 | PRF1 | CXCL11 | HNF1A |
| TRPC3 | MYCN | ZNF721 | PTPN7 | ETV4 | PAFAH2 |
| ZDHHC11 | BCHE | YWHAE | MYCN | CDH1 | FOXL2 |
| NPDC1 | MLF1IP | FOXO3 | CREB3L1 | CD52 | AQP1 |
| ACVR1B | CAPSL | DDX6 | SEMA3B | KIAA1549 | EXTL1 |
| MITF | CXCR7 | ATM | PGC | PTAFR | ACVR1B |
| PDE6B | PDZRN4 | GBP4 | CRIP3 | ABP1 | ACSL6 |
| GOLGA5 | MTMR11 | NUDC | TNFRSF11A | FOXP1 | USP44 |
| MEN1 | SCUBE2 | FANCA | SERPIND1 | PKD2L1 | SUSD1 |
| ERBB3 | F7 | TRPC3 | CDKN2B | COL1A1 | GDAP1L1 |
| TFE3 | WFS1 | TRAF1 | SLC39A8 | MLF1 | ACVR2A |
| NDUFA13 | HOOK3 | ESRP1 | MALL | ID1 | EZR |
| MLL2 | WHSC1L1 | UBE2E1 | MAEL | SLC39A5 | RHCE |
| CASP8 | GBP1 | ELK4 | ZNF521 | IL18BP | EPS15 |
| MGAM | BIRC3 | SMPD2 | CD79A | CXCR7 | RUNX1T1 |
| PHOX2B | MDFI | CDX2 | WWTR1 | JAK2 | PRF1 |
| SNX25 | ACSL1 | SDHD | PPP2R3A | CASP1 | PPP1R14D |
| WWTR1 | CECR6 | SMAD2 | MFSD7 | CDKN2B | S100A16 |
| TOX2 | C8orf42 | PDE4D | ID3 | MAP3K6 | MFSD7 |
| LCP1 | S100A5 | NRIP2 | SOCS2 | SLC30A2 | CD74 |
| PDZRN4 | WNT10B | LTV1 | NR4A3 | CANT1 | TNFRSF14 |
| CCDC80 | PGC | FCRL4 | WISP2 | ZMYM2 | KLHL34 |
| BUB1B | GSR | MTCP1 | PRKAR2B | RARRES1 | IDH1 |
| SYTL1 | PPP2R3A | GPHN | IL22RA1 | ZNF570 | POU5F1 |
| ZBTB16 | FGFR1 | BCL3 | PAX8 | FLVCR2 | SP8 |
| TLX1 | FHIT | SLC34A2 | HOXD3 | CBFA2T3 | MLLT11 |
| TMEM57 | WNT6 | MKL1 | ZBTB16 | MYB | HGFAC |
| MACF1 | ERBB2 | AHDC1 | RHOH | CCDC110 | PCSK7 |
| F5 | ZNF570 | KDR | DNASE1L2 | SERINC3 | C20orf111 |
| SLC35D2 | SEMA3B | LCP1 | ERICH1 | PLAGL1 | CLDN4 |
| PRDM1 | ZFYVE28 | IL18BP | WNT10B | RNF113A | GRM8 |
| PDLIM3 | SMPDL3B | CYTL1 | SMPDL3B | TH | ATP5I |
| ARID1A | HOXD3 | PDLIM3 | C8orf42 | ELN | YWHAE |
| CA3 | SOCS2 | MLLT6 | FBXO25 | EDNRB | MYEF2 |
| SLC22A18AS | JAKMIP1 | TCOF1 | OR4F21 | ABLIM2 | GTF3A |
| BRCA2 | WRN | WFS1 | ZNF596 | HNF1A | AIM1L |
| CTSZ | DDIT3 | GPR78 | KAT6A | ATP6V0D2 | SDC4 |
| CDK12 | FZD3 | HNRNPA2B1 | IRF4 | ACSL6 | FHL2 |
| MYOM3 | FBXO17 | MAML2 | ABP1 | BIRC3 | MTIF3 |
| FBXW7 | ETV4 | NNAT | USP44 | EVI2A | CD3D |
| SLC6A3 | TFRC | SCUBE2 | ECT2L | PLA2R1 | ADA |
| NKX6-1 | DIRAS3 | TRIM63 | CYP26B1 | PSIP1 | BTNL8 |
| BRAF | GSR | MS4A1 | IL18R1 | PRF1 | FABP1 |
| TCEA1 | CDKN2B | IL21R | PTAFR | PPP1R14D | CYTL1 |
| TNIP2 | PDZD3 | TAL2 | TBC1D10C | BCL11B | BCL2L1 |
| PRKAR1A | CREB3L1 | HOXC12 | CXCR7 | S100A16 | PAX3 |
| PSMB8 | EDNRB | ELMO3 | FAM46C | CD74 | BCL3 |
| FLVCR2 | CD274 | TNNC1 | CCDC80 | MAFB | WISP2 |
| PDK4 | WISP2 | NCOR2 | PRPH2 | KLHL34 | FGR |
| LPP | DNAH9 | PMS1 | GSR | POU5F1 | THRAP3 |
| PDGFRA | ABP1 | RCAN3 | CPZ | ETV1 | MET |
| CLTC | ADA | HOXD11 | SCUBE2 | C20orf111 | CASP5 |
| RASL11A | PDX1 | TAF15 | FCN3 | GRM8 | SOX9 |
| ASB4 | PCM1 | ARID2 | PDZRN4 | MYEF2 | SLA |
| CDKN2B | CPLX1 | PDE6B | HOXC12 | GTF3A | ELF4 |
| TLX3 | PRRX1 | C6orf48 | FZD3 | SPECC1 | DGKQ |
| CNKSR1 | GMDS | BCR | DDIT3 | CD3D | CDH11 |
| AKAP9 | PAX8 | COL1A1 | DLGAP2 | FABP1 | SH3GL1 |
| HPS1 | HEY1 | CNOT6 | TMEM45B | CYTL1 | TNFAIP3 |
| MYO1A | GPHN | ESRP2 | ARHGEF10 | TBC1D10C | TET1 |
| BRD4 | SNX25 | MYEF2 | CLN8 | CYP26B1 | SH3BP2 |
| C15orf55 | FAM46C | MED20 | HOOK3 | CD274 | RAB4A |
| CDC73 | ZNF514 | TBCC | MYOM2 | FGR | GPR56 |
| ATP6V0D2 | SPON2 | MLLT11 | S100A5 | SLA | PAN3 |
| ASXL1 | POU5F1 | ERICH1 | WRN | ARHGAP25 | TFEB |
| ZNF596 | DEFB118 | GGTLC1 | HOXC13 | FLI1 | LMO1 |
| TRERF1 | RUNX1 | STMN1 | MLF1IP | CDH11 | ESRP2 |
| WNT6 | CTSZ | HOXC13 | KBTBD11 | TRIM24 | SLC22A18AS |
| PLA2G12B | HAS1 | SESN2 | ETV1 | TET1 | ACVRL1 |
| ACVR2A | UBE2E1 | RBP2 | DIRAS3 | PLA2G12B | MLLT3 |
| CARS | KIAA1549 | TSHR | ENOSF1 | HM13 | CREB3L2 |
| PPP2R3A | ZNF521 | BTG1 | AIM1L | GPR56 | IFI6 |
| CIC | RAB42 | SS18L1 | CD79B | PAN3 | IL22RA1 |
| PCM1 | USP44 | PTPN11 | CDX2 | PDE4DIP | FRK |
| EPHX4 | TOX2 | RMI2 | PCM1 | FAM46C | SPON2 |
| EVC | RPE65 | DIRAS1 | EDNRB | MLH1 | PGC |
| PDE4DIP | IL26 | TCEA1 | ETV4 | R3HDML | NFKBIE |
| HTRA3 | PRPH2 | CNPY3 | NKX2-1 | ZNF595 | TCF7L2 |
| FLT3 | CDH11 | E2F2 | RAB42 | SMAD4 | SCUBE2 |
| KIT | CATSPER4 | ZNF572 | C1orf210 | G0S2 | PRSS8 |
| HOXA11 | LMO1 | NONO | FOXL2 | IFI6 | GMDS |
| SLC30A2 | CLIC4 | LUC7L | ACMSD | FRMD1 | CTSZ |
| IL2 | TNFAIP3 | TMEM176A | CD274 | PLAG1 | NEK3 |
| MLL3 | CIITA | DICER1 | FOXO1 | CCDC80 | HSD17B2 |
| SLC44A2 | SPECC1 | CBFB | CPLX1 | IL22RA1 | TRIM29 |
| GNMT | ENOSF1 | NR0B2 | UBE2E1 | ABHD12B | COL5A2 |
| LHFP | ZDHHC11 | ERBB3 | HTRA3 | SOCS2 | MAP7 |
| FANCF | RECQL4 | ATIC | FLVCR2 | PPP2R2B | TMEM176A |
| KRTAP19-6 | PTAFR | FAS | FHL2 | PRSS8 | CTNNB1 |
| PAQR7 | MYBL2 | MEA1 | MET | FOXO3 | RCN1 |
| ENOSF1 | CD52 | SLBP | KIAA1549 | NEK3 | PRDM1 |
| GGTLC1 | MFSD7 | ABCC5 | NEK3 | ZNF572 | GFPT1 |
| ERG | ID3 | C6orf25 | RUNX1 | DDB2 | RBP2 |
| ZNF331 | MYH11 | JAK2 | SPO11 | KIR2DL4 | GZMB |
| CTNNBL1 | TCF12 | ABP1 | MYB | TRIM29 | FUCA1 |
| AFF1 | AIM1L | MFSD10 | FGFRL1 | GPD1 | COX7C |
| FABP1 | PRF1 | MSI2 | GMDS | PRRX1 | ROS1 |
| CLN8 | MYB | CDK4 | CSMD1 | COL5A2 | PTEN |
| BCL11A | PDLIM3 | PHOX2B | GPHN | RBP2 | APOL6 |
| EBF1 | KCNE4 | CCNL1 | KCNE4 | NCKAP1L | ALK |
| PTCH1 | CCND2 | GSR | SOX9 | CA3 | MYH9 |
| CD274 | MGAM | HTRA3 | SFN | CDKN2C | WT1 |
| STK32B | C11orf52 | ALDH2 | CX3CL1 | ROS1 | ZIC1 |
| IL21R | GMDS | U2AF1 | PRRX1 | NPDC1 | JAKMIP1 |
| TCL1A | FSTL3 | CD164L2 | ALK | KLC4 | HTRA3 |
| EDAR | POLN | CX3CL1 | RMI2 | APOL6 | PRAP1 |
| CATSPERB | SLC6A3 | WWC2 | GFPT1 | ZNF141 | EPHX4 |
| SEC14L4 | PPARG | ZNF593 | ZNF570 | IKZF1 | TMPRSS2 |
| WDTC1 | CX3CL1 | SLC45A3 | MYC | FOXC1 | NFIB |
| TCF12 | ERG | HTR1D | PDLIM3 | TNNC1 | LIFR |
| PPP1R14D | LCP1 | CIITA | POU5F1 | WT1 | UBE2E1 |
| HNF4A | EVI2A | AMT | MAFB | SLC22A11 | CNKSR1 |
| MSI2 | PRDM1 | CASP8 | SNX25 | JAKMIP1 | GALE |
| FIP1L1 | SORCS2 | SDHB | PAX3 | PPP2R2C | SLC45A3 |
| SMO | CHCHD7 | MNX1 | C11orf52 | PRAP1 | BCL11A |
| ACMSD | PLAG1 | SMARCB1 | S100A16 | BCL11A | GPR39 |
